# Curation of an intensive care research dataset from routinely collected patient data in an NHS trust.

**DOI:** 10.12688/f1000research.20193.1

**Published:** 2019-08-19

**Authors:** Chris McWilliams, Joshua Inoue, Philip Wadey, Graeme Palmer, Raul Santos-Rodriguez, Christopher Bourdeaux

**Affiliations:** 1 Department of Engineering Mathematics, University of Bristol, Bristol, UK; 2 University Hospitals Bristol NHS Foundation Trust, Bristol, UK

**Keywords:** Intensive care, electronic health record, medical database, research data, critical care data, ICNARC, Philips, clinical information system

## Abstract

In this data note we provide the details of a research database of 4831 adult intensive care patients who were treated in the Bristol Royal Infirmary, UK between 2015 and 2019. The purposes of this publication are to describe the dataset for external researchers who may be interested in making use of it, and to detail the methods used to curate the dataset in order to help other intensive care units make secondary use of their routinely collected data. The curation involves linkage between two critical care datasets within our hospital and the accompanying code is available online. For reasons of data privacy the data cannot be shared without researchers obtaining appropriate ethical consents. In the future we hope to obtain a data sharing agreement in order to publicly share the de-identified data, and to link our data with other intensive care units who use a Philips clinical information system.

## Introduction

The increasing use of clinical information systems on intensive care units (ICUs) means that large amounts of patient data are being generated as part of routine care. These data are stored in electronic health records (EHR) and represent a valuable resource with huge potential to improve patient care. Collaboration between clinicians, researchers and industry stakeholders is required to realise the potential of these data by developing new methodologies and digital technologies. However, there exists a more fundamental set of barriers to making the required data available for secondary use and until these barriers are overcome the ability to maximise patient benefit via data-driven approaches will be limited. Here we introduce what we see as the four main barriers, and then explain how the publication of this data note (and its associated methodology for data curation) contributes to overcoming these barriers.

### Barrier 1: Data format

There is no standard format for storing intensive care EHR data. This is mainly due to two factors: differences between the proprietary formats used by different clinical information systems, and the high level of configurability of each system. EHR data are stored in proprietary formats designed by the companies who provide the data collection and storage software. In the intensive care units at our hospital we use the Philips ICCA clinical information system (CIS), which is currently the most widely deployed system across the NHS, with installation at 27 sites at the time of writing. Although the various available critical care CIS products do facilitate secondary data usage to some extent, they were all designed primarily as charting systems and therefore secondary use of the data is always a challenge. The main issue with ICCA is the high level of configurability of the system, meaning that data encoding can vary extensively between sites but can also change over time at a single site. The consequence of this configurability is that it can be challenging to locate and harmonise even a single simple data element, such as heart rate, for a cohort of patients over a period of time.

### Barrier 2: Data linkage

There are two related issues around data linkage: 1) different types of data from different sources within the hospital (or beyond) need to be linked in order to make the data more useful to researchers; and 2) data from different hospitals need to be combined to increase data volume and therefore statistical power.

The first issue relates to the scope of individual data sources. The ICCA database contains data collected routinely as part of patient care on ICU, but does not contain any information about what happened to the patient before or after their ICU stay. Therefore, taken in isolation, the data in ICCA are of limited use for research purposes. In order to make the data more useful they must be linked to other datasets that capture diagnoses, past medical history, outcomes etc. For this purpose we use data that is compiled locally for national audit by ICNARC (see Methods for more details). Linkage of our ICCA data to the local ICNARC data is a procedure that should be simple but is in fact challenging because of several error sources relating to the way that the data are collected. Developing a robust data linkage procedure has required an intimate knowledge of the data. Exposition of this data linkage procedure is one of the main purposes of this paper, because it will help other NHS trusts unlock secondary value from their data.

The second issue relates to the fact that individual intensive care datasets are relatively small. The general intensive care unit at UHB has 20 beds and treats around 1300 patients each year. To date the research database contains 4831 patients database and this number will increase to ~ 6100 with the update at the end of 2019. Most machine learning algorithms need more cases than this to achieve good performance, hence the motivation to link datasets across hospitals. Two US-based critical care datasets have achieved high volumes of data via different means. MIMIC-III ^1^ contains around 60,000 ICU admissions, collected from a single large teaching hospital with multiple units over a period of 12 years. Conversely the eICU database ^2^, produced by Philips, contains around 200,000 patient stays from different hospitals over a period of two years ^a^. The eICU data were collected with purpose built software to facilitate high-frequency data collection in a coherent format. Both the MIMIC and eICU datasets are publicly available and their widespread use by researchers will be hugely beneficial to patients. In the UK the CCHIC ^3^ has work on linking data from multiple hospitals with different CIS products. The challenges posed by linking data from the different proprietary systems are significant, but the data has begun to be used by researchers affiliated with the CCHIC. We feel that focusing solely on data from a single CIS system (e.g. ICCA) would significantly simplify the linkage process and that, given the widespread deployment of ICCA across the NHS, there is good potential to produce a large high-quality intensive care research database by linking data from ICCA sites only. The first stage in this process is to encourage and facilitate local pre-processing of the data at each site.

### Barrier 3: Data privacy

There is a growing consensus that the best way to unlock value from data is to share them widely and openly with researchers. Given the sensitive nature of medical data there are important ethical issues to consider in this context. However, we are ultimately of the opinion that it is unethical *not* to use routinely collected data to improve patient care. Therefore, addressing the issues around data privacy requires the development of information governance frameworks to facilitate data sharing while ensuring transparency, trust and safeguarding of patient data. The public data sharing agreements of MIMIC and the eICU represent precedents in this area that the NHS should pursue in order to unlock maximum value from their data.

In this data note we outline the steps we have taken to make our routinely collected critical care data ‘research ready’ and provide some related resources via GitHub. Our intention is that this will contribute to overcoming the above barriers, particularly by facilitating other ICUs with the ICCA system to link and process their data for secondary use. Curating our data using the methods described here has expanded our capacity for clinical reporting. We now regularly review a wide range of practices such as proning, pressure area care and prescribing. In real-time we use clinical dashboards to show the status of beds on the unit and generate retrospective reports to study trends over time. We have previously published work on the effectiveness of our clinical dashboards in improving ventilation practice via behavioural nudges ^4, 5^. Since then we have continued to expand the capabilities of the dashboards to support clinical decision making and improve the quality of care. We have collaborated with Philips on the development of dashboard intervention for acute kidney injury ^6, 7^ and have begun to explore machine learning methods for the automatic classification of ward-dischargeable patients ^8^.

In the future, under the correct information governance framework, linkage between several ICUs with ICCA could produce a large high quality critical care research dataset. In the meantime we encourage researchers to consider using our data by obtaining the appropriate ethical consents (see *Data availability*) and provide a brief summary of the data that would be available to them.

## Materials and methods

In this section we describe the processing that we have done so far to make our routinely collected data ‘research ready’. We first detail the two sources of our research data, then outline the procedure for linking data from these two sources and finally discuss the importance of further processing, including data harmonisation, to increase the general usability of these data. In the text we refer to open-source SQL and Python scripts that we have shared on our group GitHub account for readers wanting to process their own data in a similar way.

### Data sources

***ICCA.*** Philips IntelliSpace Critical Care and Anesthesia information system (ICCA) is a patient monitoring, documentation and prescribing system used in the four intensive care units at our hospital ^b^. ICCA collects rich data about a patient’s condition, both via automated data streams from bedside monitors and manually input by health care providers. These data include ventilation details, medications and regular notes from medical staff. The data are stored in a reporting database, which is managed using Microsoft SQL Server and follows a star-schema that is well documented by Philips.

The ICCA data are used by medical staff to monitor patients while they are on the unit, and secondary usage has traditionally focused on financial reporting within the trust to capture the value of care provided in each ICU stay. More recently we have started to make use of the data for clinical reporting and have established regular meetings to schedule work on reporting requests from clinicians.

***ICNARC.*** The Intensive Care National Audit and Research Centre (ICNARC) is an independent national charity set up with funding from the Department for Health and the Welsh Health Common Services Authority in 1993. The Case Mix Programme (CMP) ^9^ started in 1994 is one of ICNARC’s main national audits which today provides a comprehensive dataset across 268 critical care unit, covering 99% of all adult critical care units in the in the UK and Northern Ireland. The CMP dataset (currently version 3.1) consists of 209 data fields (as listed Table S1, *Extended data* ^10^), which overlap with most of the 34 data fields in the Critical Care Minimum dataset ^11^ and include the CCMDS subset of all 14 mandatory data fields used to generate the Healthcare Resource Group (HRG). This data is collected for every patient that passes through a CMP participating ICU and covers: basic demographic information; pre-admission details including past medical history and reason for ITU admission (using the ICNARC Coding Method); severity during the first 24 hours; number of days of organ support during their ICU stay and outcomes on both leaving the unit and then final discharge from hospital. The purpose of the audit is to provide a national resource for research and a local and national benchmarking tool for individual critical care units.

Ward Watcher ^12^ is the bespoke proprietary software (provided by Critical Care Audit Ltd) we use in the trust to collect this CMP dataset before sending it off to ICNARC. This software allows us to collect extra information for each patient that is not sent to ICNARC but is used within the Trust to generate detailed custom reports. It has been configured to automatically generate new records when a new admission is entered into a bed space on the Philips ICCA system and will pull data from the flowsheet and completed forms in ICCA for manual verification.

### Data linkage

A careful procedure is required to link datasets from different sources to produce valid and usable data. Here we describe our procedure for linking data from ICCA and ICNARC to produce patient records with both routinely collected ICU data and outcome descriptors. This method will be useful for any intensive care unit the ICCA system who want to make secondary use of their data in-house. The method is also detailed step-by-step in an iPython notebook (see Script S1, *Software availability* ^10^).

The main challenge to overcome is that erroneous entries in both datasets prevent a clean link. Without these errors the linkage would be a simple case of joining data tables on a unique identifier corresponding to each ICU stay. Therefore, we must first identify the erroneous entries and handle them according to the type of error that produced them. This procedure would not be possible without an intimate first-hand knowledge of the data and they way they are generated. There are three stages in the data linkage: first we handle the errors in the ICNARC data, then we handle the errors in the ICCA data and finally we link the two datasets together.

### Handling ICNARC errors

Every patient record in the ICNARC data ^c^ is manually validated by the data team, so we can be sure that each record corresponds to a real ICU stay and contains valid patient data. In the Ward Watcher software each ICNARC patient record links to an identifier in ICCA called the *encounterId*. In theory the *encounterId* uniquely identifies each ICU stay that has been captured in the CIS. However, there are various sources of error in the ICCA *encounterIds* which break the one-to-one mapping with patient records in Ward Watcher. For a small number of cases the patient record in Ward Watcher points to an empty or corrupt ICU stay in ICCA. In these cases we simply redirect the record in Ward Watcher to point to the correct stay in ICCA. For completeness we also create a new column to record the erroneous ICU stay that was pointed to originally.

### Handling ICCA errors

When patients are admitted to ICU, a record with a unique *encounterId* is manually created in ICCA. All data associated with that ICU stay is linked with this *encounterId* until the patient is discharged from ICU, at which point they are manually removed from the system. Since the admission and discharge actions in ICCA are conducted manually and are not retrospectively validated, there is potential for a number of different types of error. For example, patients can be admitted and discharged erroneously leading to phantom, nested or disjointed stays. All the potential types of error are listed in Table S2 ( *Extended data* ^10^), but there are broadly two classes of error, which are handled differently: 1) multiple *encounterIds* corresponding to a single ICU stay; and 2) multiple actual ICU stays with a single *encounterId*. For the first class of error, we replace the duplicate *encounterIds* with the original *encounterId* that was created for that stay such that a single coherent record is produced. We again produce a new column (specifically in the *D_Encounters* table) to record the duplicate *encounterIds* that have been replaced. For the second class of error there is no simple solution that could be robustly automated, so we leave these cases for manual processing by individual researchers ^d^. To facilitate manual processing we introduce another column (to the table *D_Encounters*) which specifies the type of error, if any, associated with each *encounterId*.

### Linking

Having handled the errors in both datasets, we now have one-to-one mapping between ICNARC records and stays in ICCA. We then extract all the CMP patient data from Ward Watcher in a standard XML format and use it to produce another table in our research database called *D_Icnarc*. This table has one row for each ICU stay and one column for each of the 209 variables in the CMP dataset, and links to other tables via *encounterId* and *ptCensusId* ^e^.

### Data harmonisation

The configurability of ICCA means that the way interventions are encoded can change over time. For retrospective studies it is necessary to search for medical concepts and variables in the SQL database, which can be time consuming. We have provided a well commented SQL script (see Script S2, *Software availability* ^10^) for locating variables in the back end of ICCA which should be useful for anyone working with the system. In general the best strategy is to search on the *longLabel* for interventions and on the *shortLabel* for the corresponding attributes, and then to calculate usage frequency to confirm that the variable located is in use. In the future we hope to produce a software tool for variable location that is usable by those without knowledge of SQL or experience of working with ICCA.

### Ethics

The full database is stored on a secure hospital server to which only UHB data managers have access. We follow the guidelines of the NHS Health Research Agency Confidentiality Advisory Group ^13^. Curation of the data for internal audit and service evaluation does not require research ethics approval, and for projects that extend beyond routine reporting we produce de-identified extracts of the required data with sensitive information removed (names, dates of birth, addresses, rare diagnoses, etc.).

## Dataset validation

The ICNARC data are validated internally at our hospital and externally at the national office. Therefore, we can have confidence in the validity of these data. The above procedure for data linkage also removes erroneous entries in the ICCA data. Users of the data must be aware that there are other sources of error in CIS data. In particular, some data are entered manually (medical notes, free form laboratory results, etc.) and are therefore vulnerable to corruption. Certain data fields are populated automatically (e.g. from bedside monitors) but not stored until a nurse confirms that the value is representative. Such fields are therefore valid when recorded but subject to missing values.

In Table 1 we provide a brief summary of 30 selected physiological variables to give readers a feel for the type of data contained in the database, including the frequency of recording of different variables and the extent of missing data values. We also provide a demographic summary of the patients represented in the data ( Table 2). Readers are referred to Supplementary Figures S1–S4 (see *Extended data* ^10^) for further demographic information, and Supplementary Figures S5–S7 for distributions of variable values.

**Table 1.** Summary of selected variables. ‘Record completeness’ is the percentage of ICU stays that contain at least one recording of the variable. ‘Frequency recorded’ is the number of times the variable is recorded per hour for the ICU stays that contain records of that variable. (Note: these frequencies are calculated over the full length of stay and so may be distorted when a variable is measured only during a subset of the stay.)

Variable	Value,mean (±1 *s*. *d*.)	Record completeness, %	Frequency recorded, mean (±1 *s*. *d*.)
Heart rate	85.88 (±19.06)	0.997	0.836 (±0.311)
GCS	10.47 (±4.75)	0.993	0.284 (±0.133)
Central Temperature	36.10 (±1.80)	0.245	0.547 (±0.666)
Peripheral Temperature	37.06 (±0.96)	0.984	0.292 (±0.123)
Respiratory rate	18.43 (±11.19)	0.996	1.310 (±0.923)
FiO2	36.50 (±14.57)	0.841	0.922 (±0.789)
PEEP	8.02 (±2.75)	0.509	0.535 (±0.387)
Airway	-	0.991	0.671 (±0.297)
pO2	10.87 (±5.71)	0.991	0.348 (±0.313)
pCO2	5.62 (±1.41)	0.991	0.350 (±0.312)
SpO2	95.71 (±3.57)	0.995	0.810 (±0.309)
Non-Invasive BP Mean	83.91 (±19.37)	0.834	0.254 (±0.367)
Non-Invasive BP Systolic	124.32 (±26.62)	0.839	0.259 (±0.364)
Non-Invasive BP Diastolic	65.87 (±18.17)	0.838	0.259 (±0.364)
Arterial BP Mean	80.04 (±18.34)	0.953	0.700 (±0.357)
Arterial BP Systolic	119.99 (±24.73)	0.954	0.698 (±0.356)
Arterial BP Diastolic	59.31 (±14.07)	0.954	0.698 (±0.356)
Serum sodium	137.27 (±5.57)	0.999	0.454 (±0.452)
Serum pH	7.40 (±0.09)	0.991	0.350 (±0.312)
Serum potassium	4.38 (±0.60)	0.999	0.451 (±0.451)
Serum ionised calcium	1.13 (±0.15)	0.991	0.351 (±0.314)
Serum bicarbonate	25.65 (±4.84)	0.991	0.622 (±0.492)
Serum urea	9.13 (±6.91)	0.991	0.107 (±0.184)
Serum creatinine	105.55 (±89.35)	0.990	0.107 (±0.184)
Bilirubin	23.92 (±48.74)	0.990	0.098 (±0.161)
Platelets	246.14 (±151.25)	0.992	0.111 (±0.337)
Haemoglobin	101.87 (±22.86)	0.991	0.109 (±0.337)

**Table 2.** Demographic summary of the cohort represented in the research dataset.

Variable	Value
Total ICU stays	4831
Gender, % female	0.396
Age, median years (IQR)	64.2 (50.8, 63.4)
LOS, median days (IQR)	2.9 (1.7, 5.4)
Readmission to ICU, # (%)	147 (3.0)
Mortality, # (%)	905 (18.7)

## Future work

The curation of this data has highlighted to us the importance of close collaboration between the people and teams responsible for collecting, administering and validating the data. The more that is known about an intensive care dataset—the way the data are collected, the way they are affected by clinical practice, idiosyncrasies in the digital systems involved, operational factors—the more value and information that can be extracted from them and ultimately the more value we can deliver to patients. In the future we will continue to improve and expand this research database. In particular we will work with colleagues in NICU, PICU and CICU to link data from the other intensive care units in our hospital. We will also look to include datasets from across the trust to capture information about patient hospital admissions outside the ICU.

We hope to work with external collaborators to develop a robust method for de-identifying medical notes. Finally, we will explore the possibility of linking with data from external NHS trusts who also use ICCA in their ICUs. Eventually the expansion of this research data will require more extensive data harmonisation to combine multiply-defined clinical concepts, and crucially will require a bespoke information governance framework to allow us to bring this data to researchers. We note that there is a precedent for such governance agreements in other projects referenced previously ^1– 3^.

## Data availability

### Underlying data

The sensitive nature of these data means that they are only available internally to UHB staff for the purposes of clinical audit and service evaluation activities via the CAG guidelines. For external researchers, ethical approval may be obtained via formal application to the NHS Integrated Research Application System (IRAS) for a specific research project. The IRAS website ( www.myresearchproject.org.uk) has full instructions; however, interested parties are advised to contact the corresponding author ( christopher.bourdeaux@uhbristol.nhs.uk) to discuss the application.

### Extended data

Zenodo: UHBristolDataScience/data-note-extended-data https://doi.org/10.5281/zenodo.3361287 ^14^.

This project contains the following extended data:

- Table S1: extended_tables/icnarc_cmp_dataset_properties.xlsx
- Table S2: extended_tables/icca_encounterid_error_types.xlsx
- Figure S1: extended_figures/admisson_types_discharge_reasons.png
- Figure S2: extended_figures/discharge_time_histograms.png
- Figure S3: extended_figures/reasons_for_admission.png
- Figure S4: extended_figures/stay_length_histograms.png
- Figures S5–S7: extended_figures/variable_hists[1-3].png

Extended data are available under the terms of the Creative Commons Attribution 4.0 International license (CC-BY 4.0).

## Software availability

**Script S1 available from:** GitHub (file ‘clean_encounterids.py’).

**Script S2 available from:** GitHub (file ‘variable_location_in_ICCA.sql’).

**Archived code at time of publication:** https://doi.org/10.5281/zenodo.3358750 ^10^.

**Licence:** MIT licence.

## Notes

^a^This is the publicly available component of the eICU dataset. The full dataset held by Philips is much larger.

^b^The use of the same database by the four units is one source of error in the data (e.g. erroneous transfers or patients being attached to the wrong unit identifier).

^c^Note that in some very rare cases there are stays which are excluded from the ICNARC data.

^d^For example, researchers may wish to simply remove such cases, although removal would likely introduce some bias since these cases usually represent readmissions to ICU. Alternatively they may wish to manually split the stay into two records.

^e^The *ptCensusId* in ICCA uniquely identifies spells in different units during the same ICU stay.
