# Supplementary material for: Curation of an intensive care research dataset from routinely collected patient data in an NHS trust
Source: F1000Res. 2019 Aug 19;8:1460. [Version 1] doi: 10.12688/f1000research.20193.1 (PMC6733376; doi:10.12688/f1000research.20193.1)
Supplement: Peer review history

# Peer review: Reviewer response for version 1

**DOI:** 10.5256/f1000research.22180.r52675

**Published:** 2019-09-03

**Authors:** Steve Harris, Min Ji Lee

**Affiliations:** 1 University College Hospital, London, UK; 2 Bloomsbury Institute of Intensive Care Medicine, University College London, London, UK

The authors describe a curated dataset of 4831 adult intensive care patients treated at the Bristol Royal Infirmary between 2015 and 2019. Two critical care data sources (ICCA and ICNARC) are linked and curated to create a single comprehensive ‘research ready’ dataset. By publishing the curation process the aim is to help external researchers make secondary use of their own routinely collected data. Fundamental barriers to making the required data available for secondary research use are discussed. Due to privacy constraints, the dataset is not fully published but external researchers may gain access through a formal application process.

 From the perspective of a novice data scientist and clinician this was an insightful and informative paper. The data note explains the rationale, barriers and methodologies allowing transparency and reproducibility for interested external researchers. Scripts outlining the dataset curation process were easy to follow with step-by-step commentary. Making this information accessible provides the opportunity for deeper understanding, in particular to those new to data science but curious about its potential. This is important given the need for close collaboration between clinicians, researchers and industry stakeholders to realise the full potential of routinely collected data to improve patient care.

 The authors discuss how the publication of the data note and curation methodology contributes to overcoming the barriers of data format and data linkage. However its role in mitigating barriers associated with data privacy is less clear. Further explanation may be of interest as the tension between maintaining data privacy and usability of data for researchers is highly relevant in this field. 

 To illustrate the barriers related to data format, the authors describe the challenges in locating and harmonising a single data element such as heart rate within the Philips ICCA clinical information system (CIS). The high level of configurability, where data elements can be renamed and relabelled between sites, can prevent cross-site collaboration and sharing of these modifications using code review despite using the same CIS. As these factors are beyond the researcher’s direct control, we would welcome the authors’ perspective on how commercial companies could make this process easier.

We confirm that we have read this submission and believe that we have an appropriate level of expertise to confirm that it is of an acceptable scientific standard.

# Peer review: Reviewer response for version 1

**DOI:** 10.5256/f1000research.22180.r52676

**Published:** 2019-08-27

**Authors:** Duncan Young

**Affiliations:** 1 Nuffield Department of Clinical Neurosciences, University of Oxford, Oxford, UK

**General comments**

 Thank you for the opportunity to review this manuscript. I have reviewed the text and figures. However I am not an expert on IT/computing and so have not commented on the details of the SQL and PYTHON code in the repositories.

 This paper describes the curation and linking of two databases containing information on patients treated on intensive care units in Bristol. The first database (ICCA) contains detailed information on patients’ stay in a single Trust’s intensive care unit collected as part of routine care. The second contains data submitted on the same patients to a national comparative audit programme (ICNARC CMP). The manuscript is well written and is clear.

 Similar challenges have been addressed elsewhere (notably by the CCHIC teams in the UK and the MIMIC-III team in the USA), though there is very little detailed information on the processes and problems these teams encountered which has been published. This paper addresses some of the lack of detail.

 The paper contains descriptions of the curation and linking processes. There are no data linked directly to the paper, as publishing identifiable patient data is not permitted. However, contact details are given to allow interested researchers to explore obtaining appropriate permissions to interrogate the data.

 In general the paper is informative and useful. It might benefit from a brief comment on how generalisable these methods are to other patient groups where highly granular data are collected such as patients treated in Emergency departments, or those undergoing surgery or invasive procedures.

**Minor detailed comments**

 The sentence on ICNARC’s origin should probably be modified to read “The Intensive Care National Audit and Research Centre (ICNARC) is an independent national charity *originally* set up with funding from the Department for Health and the Welsh Health Common Services Authority in 1993” as funding now comes from different sources.

 “Barrier 3: Data privacy”. The MIMIC-III and eICU programmes are able to share data publically and they are anonymised. There is no mention of this approach and the difficulties with true anonymisation, this paper rather assumes data will be accessed using ethical approvals.

 It might be helpful to emphasise that the XML file format that Wardwatcher software uses to export ICNARC CMP data is common to all the different software packages used to collect ICNARC data, and is not a software-specific format.

 Use of intensive care as adjective (eg “…intensive care EHR data”) is common in published papers but is probably best avoided.

**Supplementary material graphics comments**

- Discharge reasons bar chart: No X axis labels.
- Discharge time histogram: X axis labels at 5h 33m 20s intervals. Why this unusual spacing?
- Stay length: Unusual to use logged Y axis for these graphs though I assume this is because of the high frequency of single day stays.
- Variables histogram 1: FiO _2_ is fractional, not %. Units needed for heart rate, haemoglobin, respiratory rate, SpO _2_, and blood pressures on X axes.
- Variable histograms 2&3: Attention to all X axis units as above. SI notation for partial pressures (P0 _2_, PCO _2_) uses a capitalised “P”.

I confirm that I have read this submission and believe that I have an appropriate level of expertise to confirm that it is of an acceptable scientific standard.

# Response

**Published:** 2019-08-29

**Authors:** Chris McWilliams

**Affiliations:** University of Bristol, UK

Professor Young - Thank you for taking the time to read and review our manuscript. We appreciate your comments and will act on your suggestions to produce a revised version once we have received the other peer review reports. We will then also provide a detailed response to your review.
